# Autonomous reporting of ‘normal’ chest X-rays by artificial intelligence in the United Kingdom; can we take the human out of the loop?

**DOI:** 10.1093/bjrai/ubag008

**Published:** 2026-04-29

**Authors:** Katrina Nash, James Vaz, Ahmed Maiter, Christopher S Johns, Nicholas Woznita, Aditya U Kale, Abdala T Espinosa Morgado, Rhidian Bramley, Mark Hall, David Lowe, Alex Novak, Sarim Ather

**Affiliations:** Oxford Clinical Artificial Intelligence Research (OxCAIR), Oxford University Hospitals, Oxford, OX3 9DU, United Kingdom; Oxford University Clinical Academic Graduate School, University of Oxford, Oxford, OX3 9DU, United Kingdom; Radiology Department, Oxford University Hospital, Oxford, OX3 9DU, United Kingdom; Oxford Clinical Artificial Intelligence Research (OxCAIR), Oxford University Hospitals, Oxford, OX3 9DU, United Kingdom; Radiology Department, Oxford University Hospital, Oxford, OX3 9DU, United Kingdom; Department of Radiology, Sheffield Teaching Hospitals FT Trust, Sheffield, S5 7AU, United Kingdom; NIHR Sheffield Biomedical Research Centre, Sheffield, S10 2JF, United Kingdom; School of Medicine & Population Health, University of Sheffield, Sheffield, S10 2TN, United Kingdom; Department of Radiology, Sheffield Teaching Hospitals FT Trust, Sheffield, S5 7AU, United Kingdom; Imaging Department, UCLH, NW1 2BU, United Kingdom; Lungs for Living Research Centre, University College London, London, WC1E 6JF, United Kingdom; College of Medicine and Health, University of Birmingham, Birmingham, B15 2TT, United Kingdom; National Institute for Health and Care Research Birmingham Biomedical Research Centre, University of Birmingham, Birmingham, B15 2TH, United Kingdom; Institute of Translational Medicine, University Hospitals Birmingham NHS Foundation Trust, Birmingham, B15 2TH, United Kingdom; Warwick Medical School, University of Warwick, Coventry, CV4 7AL, United Kingdom; Radiology Department, University Hospitals Coventry and Warwickshire, Coventry, CV2 2DX, United Kingdom; Oxford Clinical Artificial Intelligence Research (OxCAIR), Oxford University Hospitals, Oxford, OX3 9DU, United Kingdom; Radiology Department, The Christie NHS Foundation Trust, Manchester, M20 4BX, United Kingdom; Greater Manchester Cancer Alliance, Manchester, M20 4BX, United Kingdom; Radiology Department, Queen Elizabeth University Hospital, Govan, Glasgow, G51 4TF, United Kingdom; Digital Health Validation Lab, University of Glasgow, Glasgow, G51 4TF, United Kingdom; Emergency Department, Queen Elizabeth University Hospital, Glasgow, G51 4TF, United Kingdom; Oxford Clinical Artificial Intelligence Research (OxCAIR), Oxford University Hospitals, Oxford, OX3 9DU, United Kingdom; Emergency Medicine Research Oxford (EMROx), Oxford University Hospitals, NHS Foundation Trust, Oxford, OX3 9DU, United Kingdom; Emergency Department, John Radcliffe Hospital, Headley Way, OX3 9DU, United Kingdom; Oxford Clinical Artificial Intelligence Research (OxCAIR), Oxford University Hospitals, Oxford, OX3 9DU, United Kingdom; Radiology Department, Oxford University Hospital, Oxford, OX3 9DU, United Kingdom

**Keywords:** X-rays, diagnostic radiology, artificial intelligence, intelligent systems

## Abstract

Chest X-rays (CXRs) are the most commonly performed imaging investigation. In the UK, many centers experience reporting delays due to radiologist workforce shortages. Artificial intelligence (AI) tools capable of distinguishing “normal” from “abnormal” CXRs have emerged as a potential solution. If “normal” CXRs could be safely identified and reported without human input, a substantial portion of radiology workload could be reduced.

This article examines the feasibility and implications of autonomous AI reporting of “normal” CXRs, using the United Kingdom as an example setting. Key issues include defining “normal,” ensuring generalizability across populations, and managing the sensitivity-specificity trade-off. It also addresses legal and regulatory challenges, such as compliance with IR(ME)R and GDPR, and the lack of accountability frameworks for errors. Further considerations include the impact on radiologists practice, the need for robust post-market surveillance, and incorporation of patient perspectives. While the benefits are clear, adoption must be cautious, with strong governance, legal clarity, and rigorous clinical validation to ensure safe and sustainable use.

## Why autonomous reporting?

Chest X-rays (CXRs) account for around 40% of all diagnostic imaging.^1^ Despite their clinical importance, timely reporting of CXRs is becoming increasingly difficult due to a workforce crisis. The UK currently has a 30% shortfall in radiologists, projected to worsen to 40% by 2028.^2^ Reporting backlogs and reliance on outsourcing are escalating, with an estimated 330 000 X-rays in the UK waiting over 30 days for a report.^2^^,^^3^

**Figure 1:**
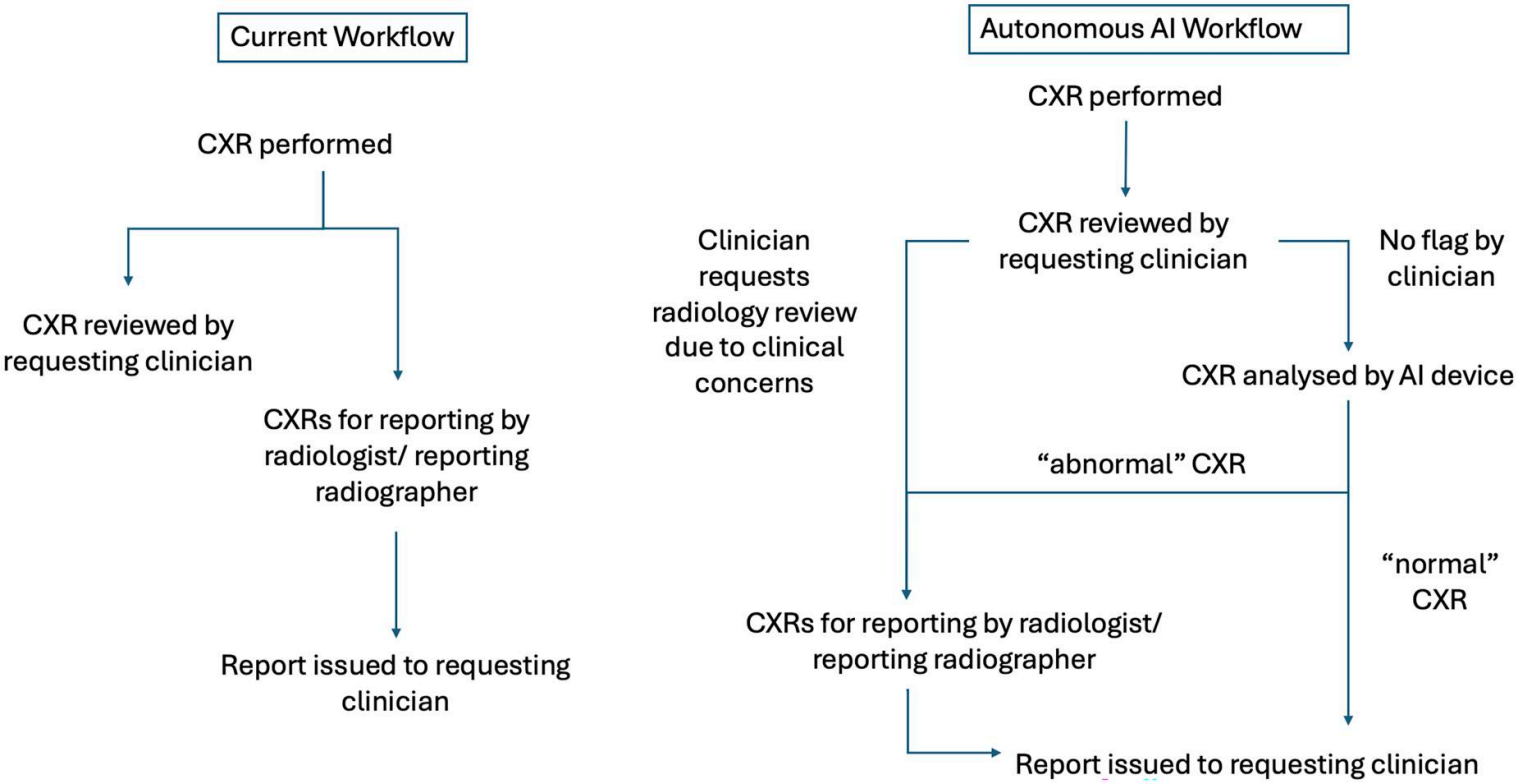
Reporting workflow for chest X-ray reporting at present, in comparison to a proposed reporting workflow with autonomous artificial intelligence deployed.

In particular, artificial intelligence (AI) reporting of “normal” CXRs, where a device classifies and reports a study as normal without human input, has emerged as a potential solution to reduce pressure on radiology departments.^4^  Figure 1 shows the current workflow in comparison to our proposed autonomous AI workflow for CXRs, where clinicians would be able to request radiology review of a CXR. Several studies show AI may accurately and rapidly distinguish “normal” from “abnormal” CXRs.^4^^,^^5^

Use of autonomous AI in clinical practice confers several advantages. Unlike humans, AI does not tire or slow down with volume, often processes images within minutes, and can interpret images 24/7. As such, AI could significantly reduce CXR reporting delays and overall radiology reporting volume, improving efficiency in radiology workflows. By offloading a large subset of “normal” CXRs, radiology reporting time can be reallocated to cases that need their expertise, such as those with subtle or complex abnormalities. Clinically, this means quicker reports for patients with normal X-rays (reducing anxiety and wait times), coupled with a faster diagnosis and treatment for those with abnormal findings, such as suspected cancer where timely diagnosis is vital.

## Clinical performance and generalizability

Successful implementation of AI devices requires the ability to perform consistently and equitably across different populations and healthcare systems. The quality and diversity of training and validation datasets are crucial for AI performance. AI devices that are poorly generalizable risk entrenching health inequalities by systematically underperforming in underrepresented groups.^6^ Devices must be robustly and prospectively evaluated across all intended populations and healthcare settings to assess real-world accuracy, workflow impact, and technical performance. It is also important to note that errors exist in human reporting. The RCR’s CXR reporting standards outline competency expectations,^7^ and we propose that any autonomous AI tool should demonstrate non-inferiority to such standards.

In clinical settings, diagnostic tools often face a trade-off between sensitivity and specificity. Sensitivity should be prioritized in autonomous deployment to minimize false negatives. Studies report sensitivities above 99% when thresholds are optimized for autonomous reporting, though this has resulted in lower specificities of (28%-67%).^6^^,^^8^ This low specificity increases false positives, potentially biasing human interpretation and leading to unnecessary investigations. These implications must be considered in pathway design.

## Definition of “normal”

The concept of a “normal” CXR lacks standardization. Interpretations may vary depending on the patient population, clinical setting and intended use; for instance, a “normal” CXR for a young patient with no chronic disease may be different to an elderly patient with chronic lung disease. In addition, in post-operative patients, it is imperative to distinguish between “expected” or “stable” post-operative findings, in comparison to those needing urgent intervention. There has been no clear consensus on this, and different studies have taken contrasting approaches. For example, one study defined a “normal” CXR as showing “no abnormal findings, including any chronic changes and/or anatomic variance”, and described “unremarkable” CXRs as those with minor, predefined or non-significant findings.^6^

Variation in this definition will have a significant impact on the volume reduction possible through autonomous reporting and clinical implications of “normal” AI reports. Therefore, for autonomous systems to function safely, a clear and consistent definition is essential. We propose that this should be agreed by professional consensus or regulatory guidance should set boundaries for the distinction of “normal”, prior to clinical implementation. In addition, manufacturers must also disclose the criteria used to define “normal” during model development and validation, to ensure correct interpretation of AI results andmeaningful comparison across studies, in addition to legal and regulatory clarity.^4^

## Regulatory, legal, and legislative landscape

Prior to deployment in the UK, AI as a medical device (AIaMD) must be approved by the Medicines and Healthcare Regulatory Agency (MHRA) designated approved body. The approval process includes review of evidence for performance and clinical safety, demonstration of risk mitigation, and adherence to data protection procedures. However, the MHRA has recently announced plans for an extensive change in programme to create clearer guidelines and standards for both premarket requirements and post market surveillance for AIaMD.^9^^,^^10^

Another key player in the UK is the Care Quality Commission (CQC), who are responsible for the Ionizing Radiation (Medical Exposure) Regulations (IR(ME)R) that govern the use of ionizing radiation in medical imaging.^11^ Importantly, the IR(ME)R currently states that every imaging study must have an identified referrer, practitioner, and operator. Currently, a human operator must act as the final signatory for reporting, meaning that implementation of autonomous reporting is currently legally prohibited in the UK.^11^ In addition, “reporting” is defined as a recorded clinical evaluation, which includes the outcome and implications; this is dependent on the clinical information and imaging request which AI generated reports may not take into account,^11^ for instance a “normal” CXR in a lifelong smoker presenting with cough and weight loss may still require follow up CT.

Notably, Oxipit was awarded the first CE mark IIb for autonomous reporting of CXR with ‘no abnormality’.^12^ This sets a precedent for future regulatory approvals and highlights the need for clear pathways to support safe deployment of autonomous systems in diagnostic workforce. We propose that stakeholders should collaborate to understand what safety thresholds and reporting considerations must be met for an amendment of IR(ME)R and to allow the human to be taken out from the system. Clear guidelines should be set out to create a regulatory pathway for autonomous AI, define safety standards required for approval, in addition to approved post-market surveillance strategies.

In addition to IR(ME)R, compliance with the UK General Data Protection Regulation (GDPR) presents another challenge. Article 22 grants individuals the right to opt out of decisions made solely through automated processing if those decisions have a legal or similarly significant effect on them, such as a clinical diagnosis.^13^ As such, provisions would need to be incorporated into clinical workflows to allow patients to opt out of autonomous reporting, which introduces additional operational complexity and must be addressed in the design of autonomous workflows.

## Ethical considerations

Ensuring equity of AI performance, avoidance of bias in the algorithms, and transparency in the use of AI in healthcare is vital.^1^ For instance, a “normal” CXR may vary by ethnicity, with some studies highlighting a lower sensitivity in minority ethnic groups. It is paramount that AI does not disadvantage underrepresented populations, leading to unequal healthcare outcomes. Fine tuning of algorithms and monitoring of algorithm performance, with specific evaluation for any AI biases, should be undertaken to avoid propagation of any health inequalities.

Accountability for errors made by AI is another important challenge. Currently, there is no established framework for the accountability of errors made by AI; instructions for use of existing commercial AI devices for radiology typically state that the healthcare professional using the device is responsible for all resulting decisions, but it is difficult to see this apply for autonomous AI. Some have argued that it should be the responsibility of the AI manufacturer, whilst others argue that it would be the hospital trust who has deployed the AI algorithm. Formulation of clear governance procedures and agreement of the legal accountability for errors encountered is paramount. Future case law will undoubtedly play an important role in clarifying liability and establishing precedent for future disputes.

## Post-deployment monitoring

AI devices require continuous monitoring to ensure ongoing safety and effectiveness, in addition to fulfilling requirements for post-deployment monitoring. AI model performance can degrade (“drift”) over time due to changes in factors such as patient population demographics, image acquisition hardware, diagnostic criteria, and reporting standards.^14^ Continuous monitoring of performance in different patient subgroups and clinical environments is therefore important for early recognition and correction of model drift. Updates should be based on data collected from real-world monitoring to ensure that the algorithm remains accurate and effective.

However, autonomous tools present unique monitoring challenges. When radiologists are no longer involved in the reporting process, traditional quality assurance methods, such as discrepancy audits, are no longer available. Therefore, mechanisms for monitoring performance and identifying critical incidences must be planned. For instance, a random selection of CXRs identified as “normal” by AI could be reported by radiologists, enabling ongoing auditing of AI performance. Reliance on reactive processes such as adverse event reporting is likely to be insufficient given the typically low rate of reporting for medical devices. However, there should be protocols in place for adverse event monitoring at both a local and national level to enable identification of any patterns in diagnostic errors made by the AI tool, in addition to any significant patient safety concerns.

## Impact on the radiology workforce

While the removal of “normal” CXRs from radiologist worklists may reduce volume, it could inadvertently increase the complexity of remaining cases. Radiologists may be left with a disproportionate number of complex or subtle findings, increasing diagnostic load, fatigue, and risk of error. There are also concerns around diagnostic calibration. Regular exposure to normal cases helps radiologists maintain confidence in calling a study “normal.” If radiologists are only exposed to abnormal cases, this calibration may shift over time, potentially impacting diagnostic thresholds. Strategies such as more frequent breaks, maintaining a mixed case list, or a random allocation of “normal” CXRs may also mitigate these risks.

Additionally, radiology trainees depend on a broad case mix to develop diagnostic proficiency. Confidently identifying a “normal” image is a critical skill and sometimes regarded as more difficult than identifying pathology. Therefore, a reduction in normal case exposure could alter learning opportunities and progression. To overcome this, we propose that registrars could have a curated normal case exposure through teaching lists, or second report cases identified as “normal” by AI.

## Patient perspective

Public trust in autonomous reporting is crucial for adoption. In a recent RCR survey, 80% of respondents supported the use of AI in radiology, yet only 5% endorsed autonomous AI.^15^ Ensuring transparency surrounding safeguards in place to prevent AI errors and ongoing monitoring processes, in addition to data protection regulations and privacy, may help to ameliorate patient concerns.

Furthermore, use of AI should be communicated to patients in an accessible manner, with clear explanations of the benefits, such as quicker report turnaround times, limitations of the relevant tool, and the standard reporting pathways used in clinical practice. Consent processes should be integrated into clinical workflows, and reports should be labelled as AI generated, giving patients the opportunity to opt-out of autonomous AI reporting and ensuring they are aware if their report has been AI generated. Nevertheless, patients must also understand who is responsible for their diagnosis and what options are available if concerns arise, for instance, how discrepancies are addressed and how further specialist opinions from a radiologist can be sought.

## Multi-professional stakeholder

In order to effectively implement autonomous reporting in the UK, we propose that a multi-stakeholder group should be established, involving the Royal College of Radiologists, the College of Radiographers, industry stakeholders, regulators, patients, and clinicians across specialties who rely on imaging to guide care. As autonomous reporting removes the safety net of a human report, it is vital to assess acceptability across all professional groups affected and ensure guidance reflects the realities of multidisciplinary clinical practice.

## Conclusion

**Figure 2:**
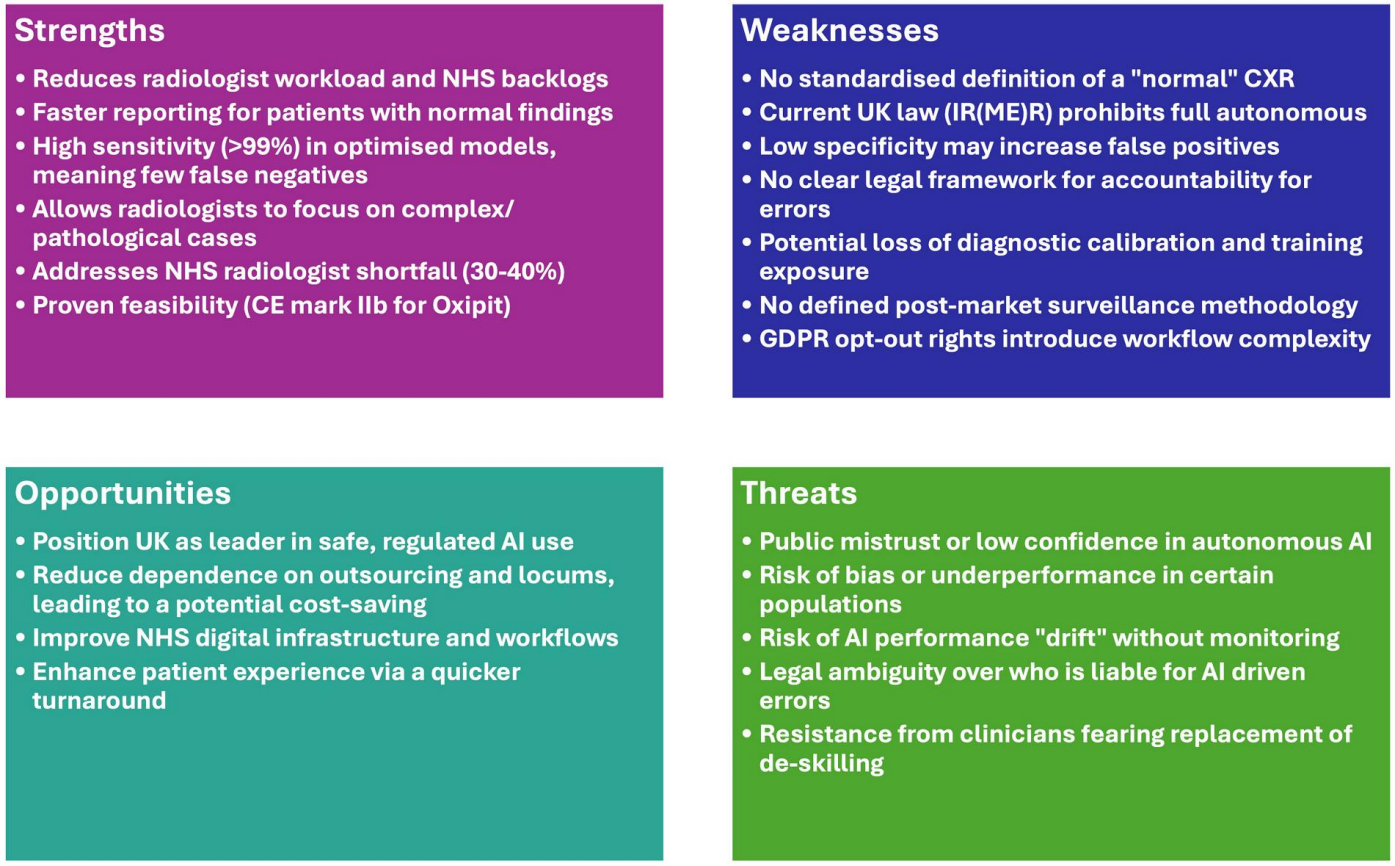
Strengths, weaknesses, opportunities, and threats (SWOT) analysis for autonomous reporting.

Autonomous reporting of normal CXRs has the potential to revolutionize healthcare by improving radiology efficiency in an era of workforce shortages. Figure 2 illustrates the key strengths, weaknesses, opportunities, and threats associated with this approach. Current evidence suggests that AI devices, such as Oxipit, which have received CE approval for autonomous use, could be used for autonomous CXR reporting in the short term to improve reporting efficiency in the UK. While this article focuses on the UK, the themes highlighted in this article have relevance across the global landscape. However, for autonomous AI to become a reality, significant steps must be taken to validate AI performance, meet regulatory requirements, clarify legal frameworks, and maintain ongoing oversight. Performance benchmarks and post-market surveillance strategies must be rigorous to ensure the highest level of patient care, and addressing the concerns of radiologists and patients will be vital for successful and responsible implementation.
